# Event-Related Potentials Reveal Preserved Attention Allocation but Impaired Emotion Regulation in Patients with Epilepsy and Comorbid Negative Affect

**DOI:** 10.1371/journal.pone.0116817

**Published:** 2015-01-14

**Authors:** Leen De Taeye, Gilles Pourtois, Alfred Meurs, Paul Boon, Kristl Vonck, Evelien Carrette, Robrecht Raedt

**Affiliations:** 1 LCEN3, Department of Neurology, Ghent University Hospital, Ghent, Belgium; 2 Department of Experimental-Clinical and Health Psychology, Ghent University, Ghent, Belgium; Radboud University Nijmegen, NETHERLANDS

## Abstract

Patients with epilepsy have a high prevalence of comorbid mood disorders. This study aims to evaluate whether negative affect in epilepsy is associated with dysfunction of emotion regulation. Event-related potentials (ERPs) are used in order to unravel the exact electrophysiological time course and investigate whether a possible dysfunction arises during early (attention) and/or late (regulation) stages of emotion control. Fifty epileptic patients with (n = 25) versus without (n = 25) comorbid negative affect plus twenty-five matched controls were recruited. ERPs were recorded while subjects performed a face- or house-matching task in which fearful, sad or neutral faces were presented either at attended or unattended spatial locations. Two ERP components were analyzed: the early vertex positive potential (VPP) which is normally enhanced for faces, and the late positive potential (LPP) that is typically larger for emotional stimuli. All participants had larger amplitude of the early face-sensitive VPP for attended faces compared to houses, regardless of their emotional content. By contrast, in patients with negative affect only, the amplitude of the LPP was significantly increased for unattended negative emotional expressions. These VPP results indicate that epilepsy with or without negative affect does not interfere with the early structural encoding and attention selection of faces. However, the LPP results suggest abnormal regulation processes during the processing of unattended emotional faces in patients with epilepsy and comorbid negative affect. In conclusion, this ERP study reveals that early object-based attention processes are not compromised by epilepsy, but instead, when combined with negative affect, this neurological disease is associated with dysfunction during the later stages of emotion regulation. As such, these new neurophysiological findings shed light on the complex interplay of epilepsy with negative affect during the processing of emotional visual stimuli and in turn might help to better understand the etiology and maintenance of mood disorders in epilepsy.

## Introduction

Patients with epilepsy have a very high prevalence of comorbid psychiatric disorders [1]. Negative affect occurs in up to 80% of patients with epilepsy [2] and may manifest as major depressive disorder (MDD) meeting the diagnostic and statistical manual IV (DSM-IV) criteria, or atypical mood disorders with waxing and waning affective symptoms called “interictal dysphoric disorder” [3] or “dysthymic-like disorder of epilepsy” [1]. In patients with epilepsy, depressive symptoms have a major negative impact on the quality of life [4–5] and increase the risk of suicide up to 10-fold [6]. Given the high impact on the quality of life and the associated elevated mortality due to suicide a better understanding of the pathogenic mechanisms of negative affect in epilepsy is important [7].

Negative affect in epilepsy has been attributed to several causes, including the psychological reaction to the chronic seizure disorder, endocrine or metabolic effects of seizures, adverse effects of antiepileptic drugs (AEDs) and common pathophysiological mechanisms between depression and epilepsy, such as neurotransmitter disturbances and abnormal frontotemporal networks [1–2, 8–10]. The common pathological changes can compromise the integrity of a functional neuronal network that is implicated in emotion control [11–13]. Emotion control refers to both early automatic forms of regulation, like controlling attention to emotional arousing stimuli, as well as higher forms of cognitive control, such as the conscious reappraisal of the emotional valence of stimuli [11]. Recently, Holtzheimer and Mayberg proposed a model for negative affect that is hallmarked by dysfunction of both forms of emotion control [14]. This model emphasizes that it is not the negative affect state that is abnormal. Instead, it is the tendency to enter the negative affect state and the inability to disengage from this state because of the impaired emotion regulation that defines mood disorders. Therefore, this study focuses on emotion control and more specifically investigates whether negative affect in patients with epilepsy is associated with dysfunction during early attention processes and/or later stages of emotion regulation.

To address this question, we used a variant of the face- or house-matching task [15], a standard task for measurement of attention and emotion regulation [16–22]. In this procedure, participants are shown a display with two houses and two faces presented in vertical and horizontal pairs. They have to attend only one pair and have to make a demanding same/different judgment on the attended pair of stimuli. The faces have either a neutral or emotional expression and are positioned either in attended or unattended spatial locations. This paradigm provides an ideal situation in which both attention and emotion can be manipulated independently [16].

Event-related potentials (ERP) are recorded during this paradigm in order to disentangle effect of attention and emotion during early (attention) and late (regulation) stages of emotion processing. One previous ERP study has investigated spatial attention during the face- or house matching task in healthy participants [17]. This study has demonstrated that the early face-sensitive N170 component amplitudes were significantly enhanced when faces were at attended spatial locations. The N170 is a negative component with latency around 170 ms that has a larger amplitude for faces than houses or other objects at occipitotemporal electrodes. The N170 has remarkable temporal and functional similarity with the vertex positive potential (VPP) that is recorded at the central midline electrode and is also typically enhanced in response to face stimuli [23–26]. Hence, it has been suggested that both N170 and VPP components are part of the same neural dipole located in or near the fusiform gyrus [27–28]. The temporally coincident N170 and VPP are the earliest markers of a reliable processing difference between faces and objects and are linked with the structural encoding of faces [17, 27]. Therefore, we measured the early face-sensitive N170/VPP components to examine whether epilepsy and negative affect have an influence on object-based attention.

Many ERP studies that study emotion have focused on a broad parietal positive component that occurs roughly 300 ms after emotional stimuli, called the late positive potential (LPP). The LPP is a robust visual ERP component that is known to have an enhanced amplitude for both positive and negative arousing emotional stimuli compared to neutral stimuli [29–33]. The magnitude of the LPP is sensitive to emotion regulation strategies and can be reduced by reappraisal of the emotional significance of stimuli, e.g. reappraising unpleasant stimuli as less negative decreases the LPP amplitude [33–35]. Hence, the LPP can be used indirectly as an electrophysiological marker of the covert processing of the emotional intensity of the visual stimuli.

In the present study, attention and emotion effects during the face- or house-matching task were compared between epileptic patients with vs. without comorbid negative affect and matched healthy controls. ERPs were used in order to explore the exact electrophysiological time course and investigate whether a possible dysfunction arises during early (attention, VPP/N170) and/or late (regulation, LPP) stages of emotion control.

## Methods

### 1 Ethics statement

The study was approved by the ethics committee of Ghent University Hospital and conducted in accordance with the declaration of Helsinki. After a full description of the procedure was provided and explained, all participants gave written informed consent prior to participation.

### 2 Participants

A total of fifty patients with refractory epilepsy were included (M/F: 26/24, mean age 34.7 years). The study took place during presurgical video-EEG monitoring in the Reference Center for Refractory Epilepsy (Ghent University Hospital, Belgium). Patients were included in the study if they met the following inclusion criteria: (i) confirmed epilepsy based on continuous video/EEG monitoring, (ii) age 18–65, (iii) Full Scale IQ score ≥ 70 on the Wechsler Adult Intelligence Scale, Third Edition (WAIS-III). Twenty-five healthy volunteers free from neurological or psychiatric symptoms were matched as closely as possible to the patients with respect to age, sex, and education (M/F: 14/11, mean age 37.0 years). The main clinical characteristics of participants are summarized in Table 1.

**Table 1:** Demographic data for each group of participants.

	**Patients with negative affect**	**Patients without negative affect**	**Controls**	**Statistics**
	**(n = 25)**	**(n = 25)**	**(n = 25)**	
BDI	23.6 (±9.4)	5.0 (±3.5)	3.6 (±3.0)	p<0.001
Age (years)	33.7 (±10.0)	35.7 (±10.9)	37.0 (±11.9)	p = 0.502
Sex (M/F)	16 / 9	10 / 15	14 / 11	p = 0.156
Education (years)	13.1 (±1.5)	13.6 (±1.7)	14.6 (±1.5)	p = 0.292
HEZ: side			NA	p = 0.683
Right	9	12		
Left	15	12		
Bilateral	1	1		
HEZ: lobe			NA	p = 0.232
Frontal	10	5		
Temporal	11	17		
Fronto-temporal	1	2		
Parietal/Occipital	3	1		
3T MRI			NA	p = 0.733
Normal	6	5		
Abnormalities	19	20		
• Frontal	7	8		
• Temporal	10	12		
∘ Medial temporal	9	10		
• Parietal/Occipital	3	2		
Epilepsy duration (years)	11.7 (±9.5)	18.9 (±10.8)	0	p = 0.016
Seizure frequency (/month)	11.9 (±12.2)	11.1 (±13.0)	0	p = 0.825
Number of AEDs (/day)	2.4 (±0.8)	2.9 (±0.9)	0	p = 0.074
AEDs total dose (mg/day)	2474.8 (±1416.1)	2631.6 (±1773.7)	0	p = 0.731
ADDs total dose (mg/day)	27.7 (±67.2)	0	0	p = 0.045
STAI State	46.3 (±10.4)	34.8 (±8.1)	28.0 (±4.9)	p<0.001
STAI Trait	52.3 (±8.3)	35.8 (±7.9)	34.6 (±7.5)	p<0.001

Values represent means (± 1 standard deviation) or numbers.

Abbreviations: *BDI* Beck Depression Inventory, *M* male, *F* female, *HEZ* hypothesized epileptogenic zone; *3T MRI* 3 tesla magnetic resonance imaging, *AEDs* antiepileptic drugs; *ADDs* antidepressant drugs, *STAI* state-trait anxiety inventory.

Presence of negative affect was assessed by using the validated Dutch version of the Beck Depression Inventory II (BDI-II) [36–37]. The BDI-II is a 21-item self-report questionnaire that assesses the severity of depressive symptomatology, including affective, cognitive, behavioral, somatic and motivational symptoms of depression. Individuals rate each symptom on a scale ranging from 0 to 3. Higher scores on the BDI reflect more negative affect with scores ranging from 0 to 63. Using the criteria proposed by Beck *et al*. (0–13 minimal, 14–19 mild, 20–28 moderate, 29–63 severe depressive symptoms) [36], a cut-off score of >14 was used to subdivide the patients in two groups: 25 patients with negative affect (mean BDI: 23.6 ± 9.4), 25 patients without negative affect (mean BDI: 5.0 ± 3.5) and 25 control participants (mean BDI: 3.6 ± 3.0). In addition, state and trait anxiety levels of all participants were measured, following standard practice, using the State-Trait Anxiety Inventory (STAI) [38], translated in Dutch [39].

### 3 Stimuli

**Figure 1:**
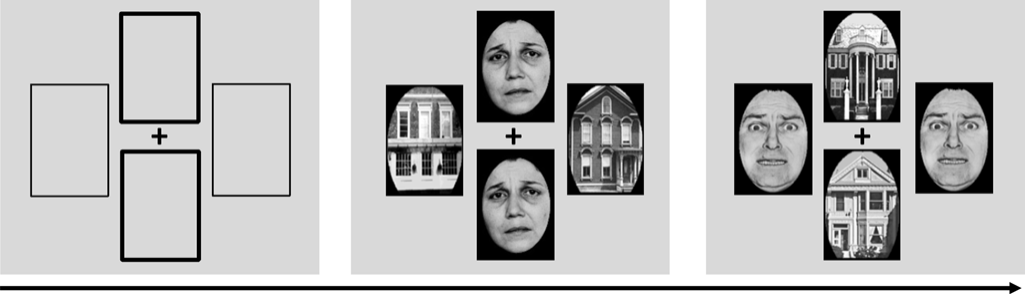
Sample visual stimuli of the face- or house-matching task. Each trial comprised a display of four pictures, with two houses and two faces arranged in vertical and horizontal pairs around a central black fixation cross. Before each block, a visual cue (i.e., thickening of two frames) instructed participants to attend either to the vertical pair or the horizontal pair of stimuli, while ignoring the other pair. Subjects had to indicate quickly and accurately whether the two stimuli at the task-relevant locations were the same or different (i.e., matching task). On any given trial, both faces had either a fearful, sad or neutral expression and were shown at task-relevant or task-irrelevant locations. The neutral, fearful and sad facial expression photographs were drawn from the set of Ekman and Friesen [40].

All stimuli comprised displays of four pictures, with two faces and two houses arranged in vertical and horizontal pairs around a central black fixation cross (Fig. 1). All pictures were black and white photographs presented on a gray background and had the same size across all experiments (108 [width] × 154 [height] pixels on a 1024 * 768 resolution screen) subtending 4.0 × 5.7° of visual angle at a 50 cm viewing distance. The stimuli included 10 fearful faces, 10 neutral faces, 10 sad faces and 20 houses, with pictures from each category repeated equally across all trials. The neutral, fearful and sad facial expression photographs were drawn from the set of Ekman and Friesen [40].

### 4 Procedure

The face- or house-matching task was adapted from previous studies [19]. Stimulus presentation and response time recording were controlled using E-Prime software 2.0 (Psychology Software Tools Inc., Pittsburgh, USA). Before each block, a 3 s display instructed subjects to attend to horizontal or vertical stimulus pairs, while ignoring the other stimulus pairs. The instruction display consisted of four empty frames placed at the location of the stimuli, with either the two horizontal or the two vertical frames being thickened. Trials began with a central fixation cross for 1 s, followed by the four-picture display for 300 ms. Subjects were asked to maintain fixation centrally throughout the trials and attend covertly to the stimulus pair at the relevant locations, in order to judge whether these two stimuli were the same or different by pressing one out of two keys. The inter-trial interval (ITI) varied randomly between 1 and 3 s. All participants completed 10 practice trials and 4 blocks of 48 trials, with two blocks where the attention was directed to horizontal positions and two blocks where the attention was directed to vertical positions. In each block, all possible combinations of two object categories (faces vs. houses), their locations, same/different identity, and facial expression were fully randomized and counterbalanced across trials, resulting in a total of 32 neutral, 32 sad and 32 fearful faces at task-relevant locations, and the same number for each expression at task-irrelevant locations (total 192 trials). Instructions emphasized both accuracy and speed. Response times were recorded from stimulus onset. Trials were excluded when there was no response within 2 seconds.

### 5 EEG recording

The electroencephalogram (EEG) was recorded with Micromed System Plus (Micromed, Mogliano Veneto, Italy) using gold electrodes placed at 27 standard locations from the extended international 10–20 system (Fp1, Fpz, Fp2, F7, F3, Fz, F4, F8, T3, C3, Cz, C4, T4, T5, P3, Pz, P4, T6, O1, Oz, O2, T9, TP9, FT9, T10, TP10 and FT10). The online reference electrode was placed on the right mastoid and ground electrode on the left mastoid. The electrocardiogram (ECG) was recorded with two ECG electrodes placed above the heart. The EEG and ECG signals were digitized online with a sampling frequency rate of 1024 Hz, anti-aliasing filter of 250 Hz, gain of 50 dB and 16 bits resolution. Electrode impedance was maintained below 10 kΩ.

### 6 ERP analysis

ERPs of interest were computed offline following a standard sequence of data transformations [41]. All offline ERP analyses were performed using BrainVision Analyzer 2 software (Brain Products GmbH, Gilching, Germany). The EEG was corrected for vertical and horizontal eye movements, blinks and ECG artifacts with an independent component analysis (ICA) that subtracts these artifact components from each electrode. The raw EEG was first decomposed into ICA components using the restricted infomax algorithm. Then the three components related to eye-movements, blinks and ECG artifacts were selected by visual inspection, relying on both the time course and the spatial maps of the components herewith generated. These components were removed and the remaining ICA components were projected back using an inverse ICA to reconstruct the artifact-free EEG. The EEG signal was then re-referenced to the average of all 27 recorded channels. The continuous EEG was first digitally filtered with a 50 Hz notch filter and a half-power band-pass filter between 0.1–30 Hz with a roll-off of 12 dB/octave. The EEG was segmented into epochs from −200 ms to +1000 ms relative to the onset of the stimuli. Baseline correction was performed on the 200 ms pre-stimulus interval and epochs with voltage exceeding ±75 µV were excluded from averaging. The average fraction of rejected epochs was 4.7% in the group of patients with negative affect, 4.1% in the group of patients without negative affect and 5.4% in the control group (one-way ANOVA F = 0.2, p = 0.8). Artifact free epochs were averaged separately for each condition and each individual. The grand average ERPs were generated by computing mean ERPs across subjects, for each condition separately. The effects of attention and emotion on sensory processing were analyzed by focusing on two well-documented ERP components: the vertex positive potential (VPP) and the late positive potential (LPP). The VPP was detected automatically as the maximum positive amplitude in the 140–210 ms interval post-stimulus onset at the central midline Cz [27]. The VPP amplitude was calculated as the mean amplitude of the 20 ms interval around this peak. The N170 measurements were made on lateral temporal-occipital electrode sites T5 and T6 using the same time window [17, 27]. The LPP amplitude was measured as the average amplitude of the 350–600 ms interval post-stimulus onset at parietal midline electrode Pz [29, 34]. Repeated measures analysis of variance (ANOVA) was used with a 2-tailed alpha level of 0.05 for all statistical tests. When assumptions of sphericity were violated (Mauchly’s sphericity test, p < 0.05), Greenhouse—Geisser epsilon correction was applied. The analyses of the ERP measures included a between-subjects factor of group (with negative affect vs. without negative affect vs. control) and a within-subjects factor of attention (task-relevant vs. task-irrelevant) and emotion (neutral vs. sad vs. fear). Post-hoc tests of simple effects were adjusted with the Bonferroni correction for multiple comparisons. In order to control for possible confounding factors of damage to the medial temporal lobe, frontal lobe and antidepressant dose, separate ANOVA’s were performed with these factors entered as covariates. Correlation between the LPP amplitude differences and antidepressant dosing were tested using 2-tailed Pearson’s correlation coefficient.

## Results

### 1 Clinical data

Differences in clinical parameters between epileptic patients with negative affect and without negative affect were assessed with unpaired t-test for continuous variables and Pearson chi-square test for categorical variables. There were no significant differences in age [t(48) = 0.7, p = 0.5], sex [Χ^2^ = 2.9, p = 0.1], years of education [t(48) = 1.1, p = 0.3], side of the hypothesized epileptogenic zone (HEZ) [Χ^2^ = 1.1, p = 0.3], lobe of the HEZ [Χ^2^ = 4.3, p = 0.2], 3T MRI abnormalities [Χ^2^ = 0.1, p = 0.7], damage to medial temporal lobe [Χ^2^ = 0.1, p = 0.8], seizure frequency [t(48) = 0.2, p = 0.8], number AEDs [t(48) = 1.8, p = 0.1] and AEDs dose [t(48) = 0.3, p = 0.7]. Duration of epilepsy was significantly higher in the group of patients without than with negative affect [t(48) = 2.5, p = 0.02]. Accordingly, longer duration of epilepsy was not associated with increased negative affect. As expected, the mean BDI score was significantly higher in the group with negative affect (23.6 ±9.4) than the group without negative affect (5.0 ±3.5) [t(48) = 9.2, p<0.001]. The STAI-S and STAI-T scores were also significantly higher in the group with than without negative affect [STAI-S: t(48) = 4.3, p<0.001, STAI-T: t(48) = 7.2, p<0.001]. Correlation analysis using a 2-tailed Pearson coefficient showed significant positive correlations of the BDI scores with the STAI-S (r = 0.73, p<0.001) and the STAI-T (r = 0.81, p<0.001) scores.

### 2 Behavioral results

**Figure 2:**
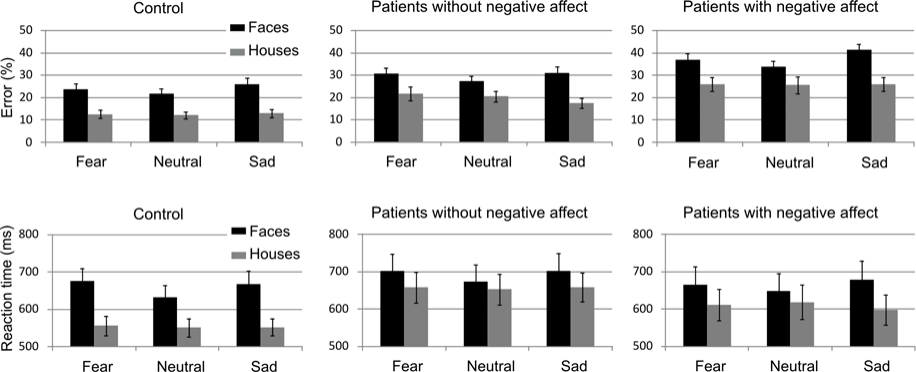
Behavioral results. Percentage errors (upper panels) and reaction time in ms (lower panels) in response to faces (black bars) and houses (grey bars), displayed separately for each group: control (left panel), patients with epilepsy without negative affect (middle) and with comorbid negative affect (right). All three groups made more errors and had slower reaction times when attended faces carried a fearful or sad emotional expression, relative to a neutral expression.

Mean response times and accuracy in same/different judgments, performed during continuous video-EEG monitoring, were computed for each subject in each of the six conditions. Behavioral results are summarized in Fig. 2. To examine data, mixed model ANOVA was performed with group as between-subject factor (control vs. patients with negative affect vs. patients without negative affect) and two within-subject factors: attention (faces vs. houses) and emotional expression (fearful vs. neutral vs. sad).

Mean error rate was 25% indicating that the matching task was relatively demanding. A significant group-related effect was found for accuracy: epileptic patients with negative affect made most errors (35%), epileptic patients without negative affect made 25% errors, while control subject had an error-rate of 18% [main effect of group F(2,72) = 10.4, p<0.001]. Subjects made more errors overall when judging faces (30%) than houses (19%) [main effect of attention F(1,72) = 103.2, p < 0.001]. Participants had lower accuracy when the faces carried a fearful or sad emotional expression [main effect of emotion F(2,72) = 4.2, p = 0.017]. Subjects made significantly more errors when the negative emotional faces were task-relevant [interaction attention*emotion F(2,72) = 7.2, p = 0.001]. Post-hoc analysis with Bonferroni correction revealed higher error rates for relevant sad [p<0.001] and fearful faces [p = 0.031] relative to neutral faces. All other comparisons were not significant. There was no significant interaction of attention or emotion with group [interaction attention*group F(2,72) = 0.3, p = 0.8, interaction emotion*group F(2,72) = 1.3, p = 0.3, interaction emotion*attention*group F(4,72) = 0.3, p = 0.9].

Analysis of reaction times (RTs) showed a mean RT of 639 ms, that was not significantly different between groups [main effect of group F(2,72) = 0.8, p = 0.4]. Participants were significantly slower to make same/different judgments with faces (672 ms) compared to houses (606 ms) [main effect of attention F(1,72) = 44.2, p<0.001]. In addition, we found a significant interaction of group with attention [F(2,72) = 4.1, p = 0.02]. In the control group there was a larger difference in RT when comparing houses (mean 554 ms) than faces (mean 659 ms) [p<0.001] than in the other two groups. RTs were significantly slower for emotional compared to neutral faces [main effect of emotion F(2,72) = 4.3, p = 0.019]. RT analysis revealed a significant interaction of attention and emotion: all subjects showed significantly slower reaction times in displays in which task-relevant faces had a fearful (681 ms) or sad (683 ms) compared to neutral (651 ms) expression [interaction attention*emotion F(2,72) = 6.7, p = 0.002]. Post-hoc analysis with Bonferroni correction revealed slower reaction times for task-relevant sad [p = 0.002] and fearful faces [p = 0.004] relative to neutral faces. All other comparisons were not significant. However, there was no significant interaction of group with emotion [interaction emotion*group F(2,72) = 0.6, p = 0.6; interaction emotion*attention*group F(4,72) = 0.6, p = 0.7].

### 3 Electrophysiological results

**Figure 3:**
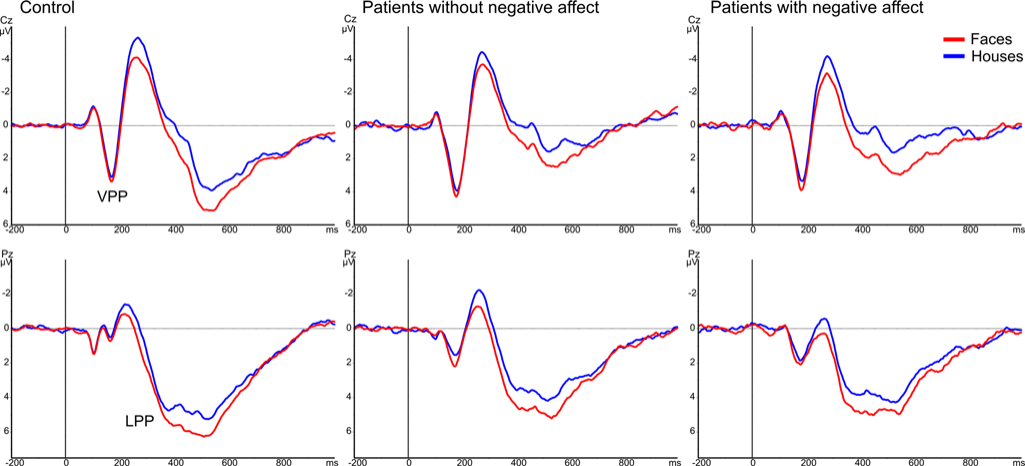
Stimulus-locked grand average ERP waveforms. Grand average ERP waveforms recorded from central midline electrode Cz (upper panels) and parietal midline electrode Pz (lower panels) in response to faces (red lines) and houses (blue lines), displayed separately for each group: control (left panel), patients with epilepsy without negative affect (middle) and with comorbid negative affect (right). In the three groups alike, the amplitude of the VPP and LPP components was significantly larger for face-cued relative to house-cued trials.

**3.1 VPP/N170.** All groups had a large face-sensitive VPP component at central midline electrode Cz (Fig. 3). The amplitude of the VPP was significantly larger on trials in which attention was focused on the face pairs relative to trials during which faces were presented outside the attention focus [main effect of attention F(1,72) = 13.0, p<0.001]. Noteworthy, there was no significant effect of group or interaction group*attention on the amplitude of the face-sensitive VPP component, indicating that the processing of the face stimuli was normal and preserved in all groups [main effect of group F(2,72) = 2.2, p = 0.1, interaction group*attention F(2,72) = 0.8, p = 0.4]. The effect of emotion or any interaction with this factor did not reach significance [F(2,72)≤1.6, p≥0.2].

The N170 is suggested to be the negative counterpart of the positive VPP, because both components are temporally coincident and have a high functional sensitivity for faces [27]. This is confirmed by our ERP results showing very similar effects of attention for the VPP and N170 components. The amplitude of the N170 at the T5 and T6 electrodes was significantly larger when faces were attended compared to when houses were attended [main effect of attention F(1,72) = 5.4, p = 0.023 at T5 electrode and F(1,72) = 22.1, p<0.001 at T6 electrode]. However, not all subjects showed a clear N170 component, because there were more artifacts and noise at the lateral T5 and T6 electrodes than at the central midline electrode Cz. Therefore, we focus on the VPP component in this study.

**Figure 4:**
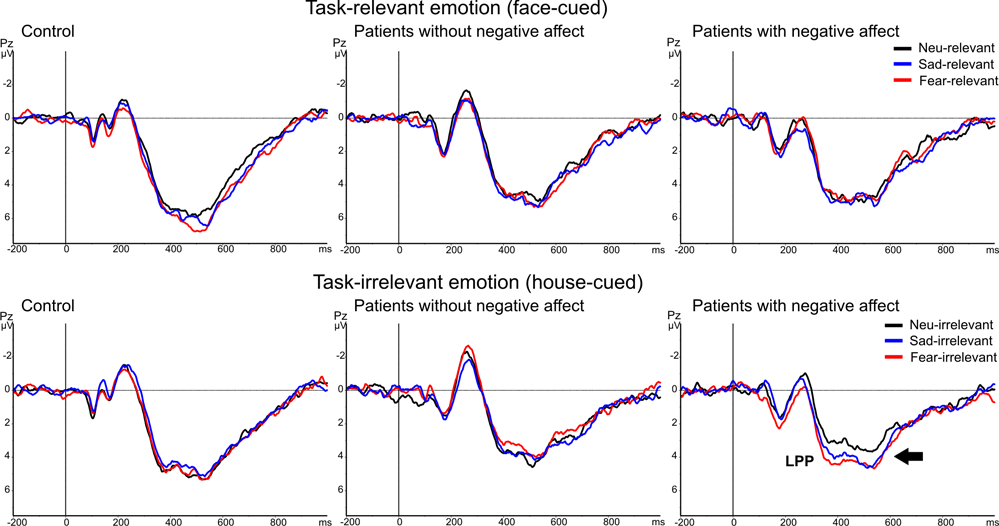
LPP results. Grand average ERP waveforms recorded from parietal midline electrode Pz in responses to task-relevant faces (upper panels) and houses (lower panels), displayed separately for each group: control (left), patients with epilepsy without negative affect (middle) and with comorbid negative affect (right). Note that only in the group of epileptic patients with negative affect, when the emotion was task-irrelevant, the amplitude of the LPP was significantly larger for sad and fearful expressions compared to neutral faces.

**3.2 LPP.** The emotion-sensitive LPP component was analyzed at the parietal midline electrode Pz from 350 to 600 ms post stimulus (Fig. 4). There were no significant differences between groups on the mean amplitude of the LPP [main effect of group F(2,72) = 1.7, p = 0.2]. In all subjects the amplitude of the LPP component was enhanced for displays in which faces were task-relevant compared to displays in which houses were task-relevant [main effect of attention F(1,72) = 49.7, p<0.001] (Fig. 3). In addition, we found a significant three-way interaction of group with attention and emotion [F(4,72) = 3.0, p = 0.021]. For post-hoc analyses we made pairwise comparisons with Bonferroni correction between the 3 emotional conditions (fear vs. neutral vs. sad) within each attention condition (house vs. face) and within each group. In total 18 comparisons were made (3 emotion * 2 attention conditions * 3 groups). These analyses revealed that only in patients with negative affect the LPP component was significantly increased in response to task-irrelevant sad [p = 0.026] and fearful faces [p = 0.003] relative to task-irrelevant neutral faces. All other comparisons were not significant.

Correlation between the differences of the LPP amplitudes between unattended emotional and neutral faces and BDI and STAI scores were tested using 2-tailed Pearson’s correlation coefficients. There were significant positive correlations found between the LPP amplitude differences between unattended fearful and neutral faces and the BDI (r = 0.34, p = 0.014) as well as the STAI (r = 0.29, p = 0.038). No such significant correlations were found between the LPP amplitude differences between unattended sad and neutral faces and either the BDI (r = 0.20, p = 0.155) or the STAI (r = 0.14, p = 0.335).

The type and location of the lesions in each group turned out to be very heterogeneous (S1 Table). Most common lesions were hippocampal sclerosis, focal cortical dysplasia, cysts and cavernomas. Only one patient had a brain tumor, which was located in the left posterior hippocampus and suspected to be a low-grade glioma. This patient was part of the group of patients with epilepsy without negative affect. In order to examine whether damage to the medial temporal lobe has an influence on the emotion modulation of the LPP, all patients with epilepsy, regardless of negative affect, were subdivided into two groups: one group where the 3T MRI showed clear damage to the medial temporal lobe (n = 19) and one group without damage to the medial temporal lobe (n = 31). Noteworthy, repeated-measures ANOVA showed no significant main effect of medial temporal lobe damage on the LPP amplitude [F(1,48) = 1.3, p = 0.3] and there were no significant interactions with other factors. In addition, a separate analysis that examined the effect of damage to the frontal lobe showed neither significant main effect of frontal lobe damage on LPP amplitude [F(1,48) = 0.4, p = 0.5] nor significant interactions with other factors. These results suggest that damage to the medial temporal lobe or frontal lobe did not account for the amplitude modulation of the LPP.

Another possible confounding factor is that some patients of the group with negative affect took antidepressant drugs (ADDs) while none of the participants in the other two groups took ADDs. However, there was no significant main effect of ADD dose on the LPP [F(1,23) = 0.02, p = 0.9] and no significant interactions of the ADD dose with the other experimental factors [attention*ADD dose F(1,23) = 3.1, p = 0.1; emotion*ADD dose F(2,23) = 1.8, p = 0.2; attention*emotion*ADD dose F(2,23) = 0.4, p = 0.7]. Moreover, a correlation analysis using a 2-tailed Pearson coefficient failed to show a significant association between the ADD dose and the amplitude difference between unattended fear faces compared to unattended neutral faces [r = 0.10, p = 0.62] or unattended sad faces compared to unattended neutral faces [r = 0.14, p = 0.50].

## Discussion

This study provides novel neurophysiological findings on the processing of emotional stimuli in patients with epilepsy with and without comorbid negative affect, when compared to a group of matched healthy controls. At the behavioral level, all subjects made more errors and had slower reaction times when attended faces carried a fearful or sad emotional expression, relative to a neutral expression. These results suggest that negative emotional face expressions, when attended, interfered with the matching task requiring the processing of the identity (as opposed to emotional content) of the face stimuli. At the electrophysiological level, the face-sensitive VPP had enhanced amplitude for attended faces compared to houses, equally so in all three groups and regardless of the emotional content of the face stimulus. These ERP results indicate that attention was directed to the correct stimulus category independently of the emotional content of the face, and that this early structural encoding of faces was normal and preserved in patients with epilepsy, regardless of negative affect. By contrast, the amplitude of the LPP was significantly enhanced for negative emotional expressions when faces were unattended, but only in patients with comorbid negative affect. The modulation of the LPP component by unattended emotional stimuli during the late stages of stimulus processing suggests that emotion regulation is disturbed in patients with epilepsy and comorbid negative affect.

Our behavioral results show that in all groups the attended negative emotional face expressions decreased task performance, which resulted in lower accuracy and slower reaction times, compared to attended neutral faces. These findings are in line with previous studies that have reported interference effects created by negative emotional stimuli [16, 20, 42]. From an evolutionary perspective, priority processing of emotional information facilitates adaptive behavior, promoting survival and reproductive success [43–44]. The enhanced processing demands associated with emotional stimuli leave limited resource capacities for performance during the task, that requires to match the identity of the two attended visual stimuli [42, 45–46]. Accordingly, these behavioral findings confirm that in all three groups, emotion interfered with task performance when it was attended, although not explicitly task-relevant.

The results obtained for the VPP/N170 component reveal a clear gating effect, in the expected direction, of object-based attention mechanisms. All subjects showed enhanced amplitudes of the VPP/N170 components for attended faces (regardless of their emotional content) compared to attended houses. Our findings are in agreement with a previous ERP study using the same task in healthy adult participants that reported similar increased amplitudes of the N170 in response to attended faces [17]. Importantly, in our study, this object-based attention effect was evidenced in all three groups alike; suggesting that neither epilepsy alone, nor epilepsy combined with negative affect actually impaired the normal and early structural encoding of faces. According to previous ERP studies, [24–27], the VPP is the counterpart at the vertex of the occipito-temporal N170 component and this dipolar activity reflects the earliest markers of a reliable processing difference between faces and objects. Therefore, our new ERP findings clearly show that this early categorization process is spared in epilepsy with or without negative affect.

By contrast, at a later time point following stimulus onset than the VPP, we found evidence for a modulatory effect of epilepsy with comorbid negative affect on the processing of these complex stimuli. We found that in patients with epilepsy and comorbid negative affect the amplitude of the LPP was significantly modulated when the emotional faces were unattended. It seems contradictory that emotional stimuli presented outside the focus of attention have a stronger influence on the LPP than when the same stimuli are attended. This is in contrast with many ERP studies that have shown that the LPP component has enhanced amplitudes for attended negative emotional stimuli compared to neutral stimuli in healthy participants [29–35]. However, these studies had longer picture presentation time (≥1 second) and the emotional expression of the face was task relevant because participants had to rate pictures for arousal and valence. Therefore, the task-relevant emotional content was probably much more strongly processed, reflected by increased LPP amplitudes. In our study, the stimuli were presented very briefly (300 ms) and the emotional expression was not explicitly relevant for the matching task. This might explain why in healthy control group and the group of patients without negative affect there was no significant modulation of the LPP by the emotional expression of the faces. By contrast, the LPP was enhanced in patients with epilepsy and comorbid negative affect when the emotional faces were unattended. It is probable that the negative affect triggers an automatic emotional processing or vigilance effect (reflected by increased amplitude of the LPP) when negative stimuli are distracters. This could point to a deficit to inhibit distracting negative emotional information, or conversely, to a better sensitivity to process them “covertly” outside the focus of attention. Accordingly, our LPP results show that negative emotional distracters have an influence on the late stages of stimulus processing in epilepsy patients with negative affect. Hence, the deficit in these patients is a deficit during the late stages of emotion control, during which they fail to ignore distracting emotional information, unlike the two other groups where the late processing of visual stimuli is not influenced by emotional distracters at unattended locations.

A few limitations have to be pointed out. Firstly, we have not included an additional control group of patients with negative affect but without epilepsy. More than 70% of mood disorders in epilepsy are atypical and fail to meet any of the diagnostic criteria of the DSM-IV [1, 3]. Therefore, a group of patients with mood disorders would not fully control for the type of negative affect in epilepsy. Patients with major depressive disorder, for example, would have more severe depression than the patients included in this study. As expected, epileptic patients with negative affect were not only showing higher levels of depressive symptoms (BDI) than patients without negative affect, but also higher levels of anxiety (STAI). This multicollinearity is not surprising, but instead in line with previous studies that have identified comorbid anxiety symptoms in 73% of patients with epilepsy and depression [7]. There is a substantial symptom overlap and comorbidity between depression and anxiety and both disorders are characterized by high levels of negative affect [47]. Accordingly, future ERP studies are needed in order to establish whether depression or rather anxiety lies at the root of the emotion regulation disorder observed in our study at the level of the LPP. To the best of our knowledge, until now no ERP studies have been published on the face- or house-matching task in patients with negative affect. Studies on the VPP and LPP in mood and anxiety disorders during other tasks have yielded mixed results, depending on the used paradigm, type of stimuli, stimulus presentation time and study population. There is evidence for increased processing of negative emotional stimuli soon after stimulus presentation, reflected by enhanced early ERPs like the VPP, followed by avoidance of unpleasant stimuli at later processing stages, reflected by a reduced LPP for aversive stimuli, both in patients with general anxiety disorder [48] and patients with major depressive disorder [49]. In contrast, other studies have reported increased LPP for aversive compared to neutral pictures among subjects with high negative affect [50–51]. Therefore, it would be very interesting in future studies to compare ERP results during the face-or house-matching task in different control groups of patients without epilepsy but with other types of negative affect disorders, such as major depressive disorder, dysthymic disorder, bipolar disorder, general anxiety disorder, in order to evaluate whether this is a general effect found across these negative affect disorders, or instead, whether it is specific for negative affect in epilepsy.

Secondly, the administration of antidepressant drugs (ADDs) in one group selectively but not in the two others may have obscured our new ERP findings. However, if ADDs would influence the amplitude of the LPP, then we would expect to see a main effect, and not a complex three-way interaction effect, as we report here. Moreover, when the ADD dose was added as a covariate in the statistical analysis for the LPP, no significant contribution of this factor was found. Furthermore, there was no significant correlation between the ADD dose on the one hand and the LPP amplitude differences between unattended sad or fearful compared to unattended neutral faces on the other hand. Taken together, it therefore appears very unlikely that the condition-specific modulation of the LPP might be explained by exposure to antidepressant medication.

Thirdly, we did not use eye tracking during the face- or house-matching task. However, participants were asked to maintain central fixation and eye movements were discouraged and unlikely with this specific demanding matching task, given the brief stimulus duration and task requirements used. This was formally confirmed by previous studies with eye tracking during the same task that demonstrated that saccades were very rare, with no major differences in eye position associated with the experimental factors [15–16, 18–19]. In addition, an ICA was used to correct for horizontal and vertical eye movements.

Fourthly, it is important to consider the type of lesions because specific lesions can cause widespread reorganization of neural networks. For example, patients with left sided tumors show signs of functional reorganization and employ a much broader bilateral network during language processing than healthy controls [52]. However, in our study, only one patient had a brain tumor in the group of patients without negative affect. The type, size and location of lesions of the patients were very heterogeneous and therefore the groups for each lesion were too small in size to compare at the statistical level the possible differential effect of each specific type of underlying lesion on negative affect and ERP results. Notwithstanding this limitation, we note that it may be possible that some of these lesions could have had a greater influence on our ERP results than other ones, especially lesions located in regions that are presumably important for emotion control processes, like the frontal and medial temporal regions. Therefore, we performed additional data analyses and grouped the patients based on the presence of either frontal or medial temporal lobe structures lesions, but these analyses failed to show any significant effect on the LPP amplitude. Hence, the abnormalities arising during the later stages of emotion stimulus processing in patients with epilepsy and comorbid negative affect could not be linked to damage in one specific lobe but are more likely the result from dysfunction in a broad network for emotion control in which both cortical and subcortical structures interact with each other [11–12].

Comorbid negative affect in patients with epilepsy has often been considered to be a consequence or complication of the chronic seizure disorder. However, a fascinating bidirectional relationship between epilepsy and depression has recently been demonstrated [10, 53]: not only are patients with epilepsy at greater risk of developing a depressive disorder, but patients with primary depressive disorders are at greater risk of developing epilepsy [53–54]. This suggests that the pathogenic mechanisms may be strongly intertwined and the structural and functional alterations from one disease are likely to trigger the other [8, 55]. Identification of these common underlying pathogenic mechanisms may shed new light on the neurobiological bases of mood disorders and epilepsy. Our findings suggest comorbid negative affect in patients with epilepsy may be due to impaired emotion regulation.

In conclusion, the face-sensitive VPP results indicate that early attention was allocated to the correct stimulus category and that early stimulus processing was preserved in all patients with epilepsy, regardless of negative affect. Conversely, the LPP results suggest that during later stages of stimulus processing the emotion regulation is disturbed, but only in patients with epilepsy and comorbid negative affect. These new neurophysiological findings shed light on the complex interplay of epilepsy with negative affect during the processing of emotional visual stimuli and in turn might help to better understand the etiology and maintenance of mood disorders in epilepsy.

## Supporting Information

S1 Table Raw data of the 75 individual participants with demographic characteristics, MRI lesion descriptions, behavioral results and ERP component amplitudes. (XLSX)
